# Cellular prion protein promotes post-ischemic neuronal survival, angioneurogenesis and enhances neural progenitor cell homing via proteasome inhibition

**DOI:** 10.1038/cddis.2015.365

**Published:** 2015-12-17

**Authors:** T R Doeppner, B Kaltwasser, J Schlechter, J Jaschke, E Kilic, M Bähr, D M Hermann, J Weise

**Affiliations:** 1 Department of Neurology, University of Duisburg-Essen Medical School, Essen, Germany; 2 Regenerative and Restorative Medical Research Center, Istanbul Medipol University, Istanbul, Turkey; 3 Department of Neurology, University of Jena, Jena, Germany; 4 Department of Neurology, University of Goettingen, Goettingen, Germany; 5 HELIOS Clinic Plauen, Department of Neurology, Plauen, Germany

## Abstract

Although cellular prion protein (PrP^c^) has been suggested to have physiological roles in neurogenesis and angiogenesis, the pathophysiological relevance of both processes remain unknown. To elucidate the role of PrP^c^ in post-ischemic brain remodeling, we herein exposed PrP^c^ wild type (WT), PrP^c^ knockout (PrP−/−) and PrP^c^ overexpressing (PrP+/+) mice to focal cerebral ischemia followed by up to 28 days reperfusion. Improved neurological recovery and sustained neuroprotection lasting over the observation period of 4 weeks were observed in ischemic PrP+/+ mice compared with WT mice. This observation was associated with increased neurogenesis and angiogenesis, whereas increased neurological deficits and brain injury were noted in ischemic PrP−/− mice. Proteasome activity and oxidative stress were increased in ischemic brain tissue of PrP−/− mice. Pharmacological proteasome inhibition reversed the exacerbation of brain injury induced by PrP−/−, indicating that proteasome inhibition mediates the neuroprotective effects of PrP^c^. Notably, reduced proteasome activity and oxidative stress in ischemic brain tissue of PrP+/+ mice were associated with an increased abundance of hypoxia-inducible factor 1*α* and PACAP-38, which are known stimulants of neural progenitor cell (NPC) migration and trafficking. To elucidate effects of PrP^c^ on intracerebral NPC homing, we intravenously infused GFP^+^ NPCs in ischemic WT, PrP−/− and PrP+/+ mice, showing that brain accumulation of GFP^+^ NPCs was greatly reduced in PrP−/− mice, but increased in PrP+/+ animals. Our results suggest that PrP^c^ induces post-ischemic long-term neuroprotection, neurogenesis and angiogenesis in the ischemic brain by inhibiting proteasome activity.

Endogenous neurogenesis persists in the adult rodent brain within distinct niches such as the subventricular zone (SVZ) of the lateral ventricles,^1, 2, 3, 4^ which host astrocyte-like neural stem cells and neural progenitor cells (NPCs). Focal cerebral ischemia stimulates neurogenesis, and NPCs proliferate and migrate towards the site of lesion where they eventually differentiate.^5, 6, 7^ In light of low differentiation rates and high cell death rates of new-born cells,^6, 8, 9^ post-stroke neurogenesis is scarce.^10^

Cellular prion protein (PrP^c^) is a glycoprotein that is attached to cell membranes by means of a glycosylphosphatidylinositol anchor.^11^ Although PrP^c^ is ubiquitously expressed, it is most abundant within the central nervous system. Conversion into its misfolded isoform PrP^sc^ causes neurodegenerative diseases such as Creutzfeldt-Jacob disease.^11, 12^ While a large body of studies analyzed the role of PrP^sc^ in the context of transmissible spongiform encephalopathies, little is known about the physiological role of PrP^c^. Studies performed during both ontogenesis and adulthood suggest that PrP^c^ regulates neuronal proliferation and differentiation, synaptic plasticity and angiogenesis.^13, 14, 15, 16, 17, 18^ The role of these processes under pathophysiological conditions, however, is largely unknown.

Previous reports suggested a role of PrP^c^ in post-ischemic neuroprotection.^19, 20, 21, 22, 23, 24^ Thus, PrP^c^ was found to be overexpressed in ischemic brain tissue.^19, 20, 21, 22, 23, 24^ PrP^c^ deficiency aggravated ischemic brain injury, possibly via enhanced ERK-1/2 activation and reduced phosphorylation of Akt, thus ultimately culminating in increased caspase-3 activity,^21, 24^ whereas PrP^c^ overexpression protected against ischemia.^19, 20, 21, 22, 23, 24^ Nevertheless, these studies focused on acute injury processes with a maximal observation period of 3 days, leaving the biological role of PrP^c^ in post-stroke neurogenesis and angiogenesis unanswered. To clarify the role of PrP^c^ in the post-acute ischemic brain, we herein exposed PrP^c^ wild type (WT), PrP^c^ knockout (PrP−/−) and PrP^c^ overexpressing (PrP+/+) mice to focal cerebral ischemia induced by intraluminal middle cerebral artery (MCA) occlusion, evaluating effects of PrP^c^ on neurological recovery, ischemic injury, neurogenesis and angiogenesis, as well as the homing and efficacy of exogenously delivered NPCs.

## Results

### PrP^c^ ameliorates post-stroke neurological impairment and induces long-term neuroprotection

**Figure 1:**
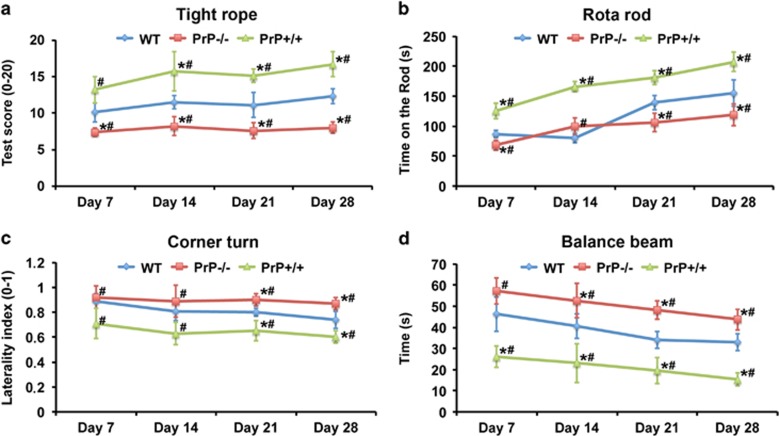
Cellular prion protein (PrP^c^) ameliorates post-ischemic neurological impairment. (**a**) Tight rope, (**b**) rota rod, (**c**) corner turn and (**d**) balance beam test in wild type (WT), PrP^c^ knockout (PrP−/−) and PrP^c^ overexpressing (PrP+/+) mice exposed to 45 min of MCA occlusion followed by 28 days reperfusion. *Significantly different from WT mice, *P*<0.05. ^#^Significantly different from PrP−/− or PrP+/+ mice, *P*<0.05

**Figure 2:**
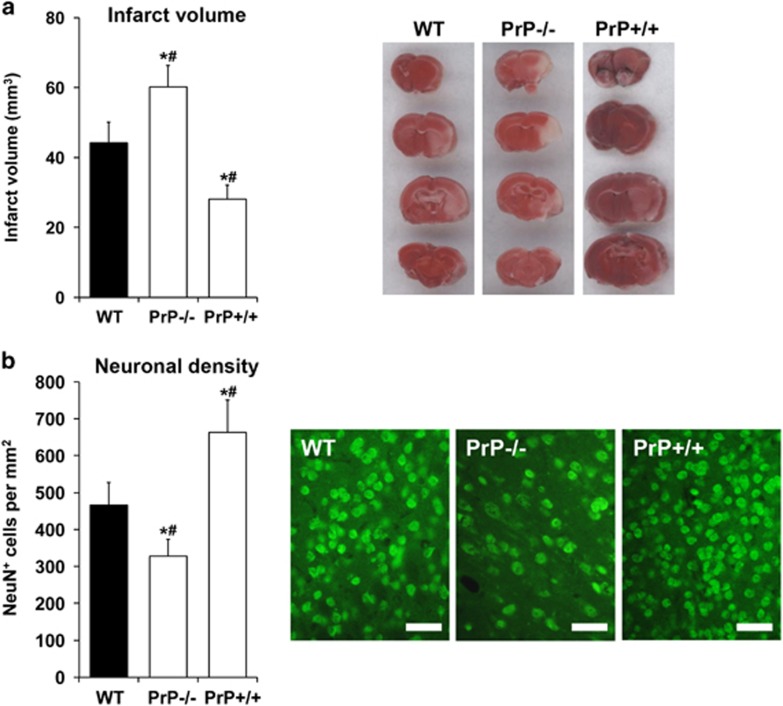
PrP^c^ induces sustained neuroprotection after focal cerebral ischemia. (**a**) Infarct volume determined by 2,3,5-triphenyltetrazolium chloride (TTC) staining and (**b**) neuronal density determined by NeuN immunohistochemistry in the striatum in WT, PrP−/− and PrP+/+ mice exposed to 45 min MCA occlusion followed by 24 h (in **a**) or 28 days (in **b**) reperfusion. Representative photographs are also shown, which in (**b**) were taken in the core of the ischemic striatum. Scale bars: 20 *μ*m. *Significantly different from WT mice, *P*<0.05. ^#^Significantly different from PrP−/− or PrP+/+ mice, *P*<0.05

Before analyzing effects of PrP^c^ on both neurogenesis and underlying mechanisms, we first assessed whether or not PrP^c^ induces sustained reduction of motor coordination impairment and histological brain injury. Using the tight rope, rota rod, corner turn and balance beam test, assessment of motor coordination for as long as 28 days after 45 min MCA occlusion revealed significantly improved neurological recovery in PrP+/+ mice when compared with both WT mice and PrP−/− mice (Figure 1). On the contrary, PrP−/− mice showed significantly reduced neurological recovery not only when compared with PrP+/+ mice but also in comparison with WT mice (Figure 1). In line with better test performance of PrP+/+ mice, PrP+/+ mice showed significantly reduced post-ischemic brain injury when compared with WT mice and PrP−/− mice (Figure 2). Again, PrP−/− mice did not only show increased brain injury when compared with PrP+/+ mice, but also developed significantly larger brain injury in comparison with WT animals (Figure 2). Of note, less than one percent of the total amount of NeuN^+^ cells shown for the analysis of late brain injury (Figure 2b) co-label with BrdU (e.g., 0.53% cells in the PrP−/− group), thus indicating that the total amount of NeuN^+^ cells depicted in Figure 2b depends on neuronal density rather than on neurogenesis.

### Post-ischemic neurogenesis and angiogenesis are increased by PrP−/− and PrP+/+

**Figure 3:**
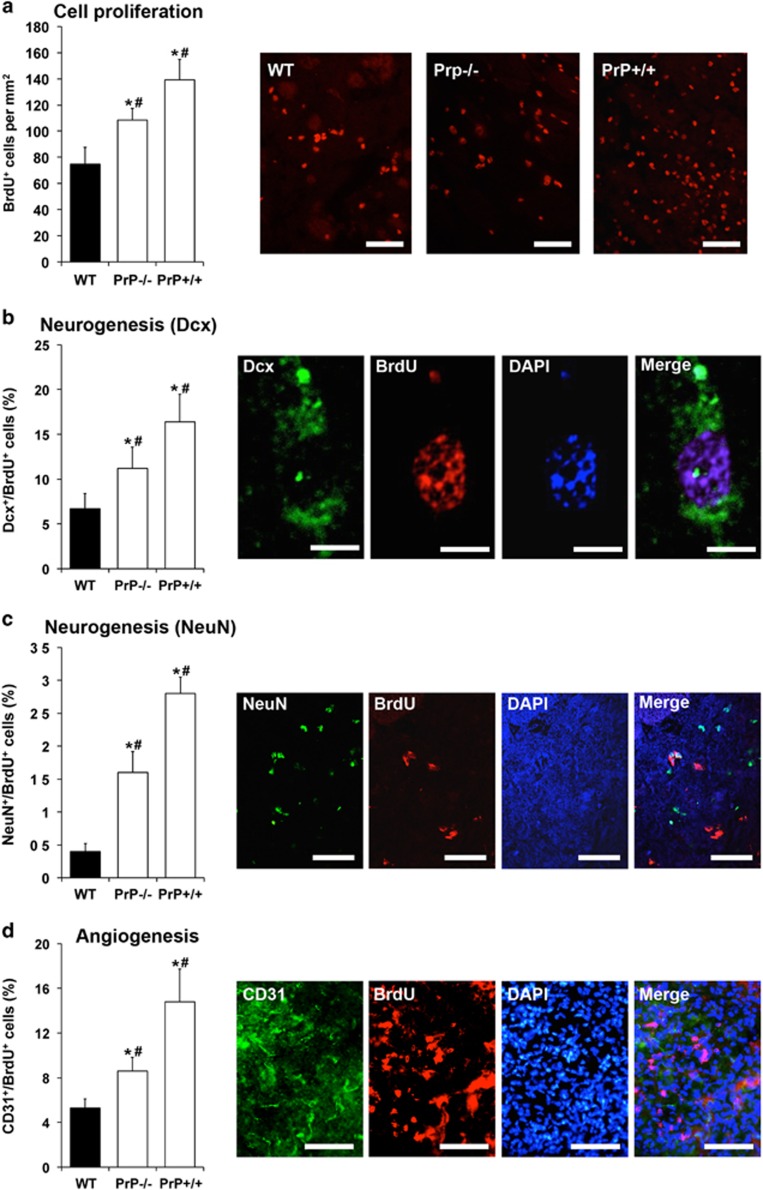
Post-ischemic neurogenesis and angiogenesis are increased in PrP−/− and PrP+/+ mice. (**a**) Cell proliferation assessed by bromodeoxyuridine (BrdU) immunolabeling, (**b**) neurogenesis evaluated by co-labeling of the immature neuronal marker Dcx and BrdU, (**c**) neurogenesis determined by co-labeling of the mature neuronal marker NeuN and BrdU and (**d**) angiogenesis examined by co-labeling of the endothelial marker CD31 and BrdU in the striatum of WT, PrP−/− and PrP+/+ mice exposed to 45 min MCA occlusion followed by 28 days reperfusion. Representative photographs are presented from the core of the ischemic striatum from PrP+/+ mice. Scale bars: 50 *μ*m. *Significantly different from WT mice, *P*<0.05. ^#^Significantly different from PrP−/− or PrP+/+ mice, *P*<0.05

We next analyzed post-ischemic neurogenesis and angiogenesis on day 28 post-stroke. Analysis of BrdU^+^ cells within the ischemic lesion revealed significantly more BrdU^+^ cells in PrP−/− than in WT mice (Figure 3a). Interestingly, PrP+/+ mice exhibited even more BrdU^+^ cells; their number significantly exceeded that in WT mice and PrP−/− mice (Figure 3a). Analysis of differentiation patterns of BrdU^+^ cells revealed significantly increased co-expression with Dcx, NeuN and CD31 both in PrP−/− and in PrP+/+ mice (Figures 3b and d). Again, PrP+/+ mice showed significantly higher figures than PrP−/− mice (Figures 3b and d). These data suggest that both post-ischemic neurogenesis and angiogenesis are increased in PrP−/− mice and in PrP+/+ animals.

### Enhanced post-ischemic neurogenesis and angiogenesis in PrP−/− mice is a consequence of increased brain injury

**Figure 4:**
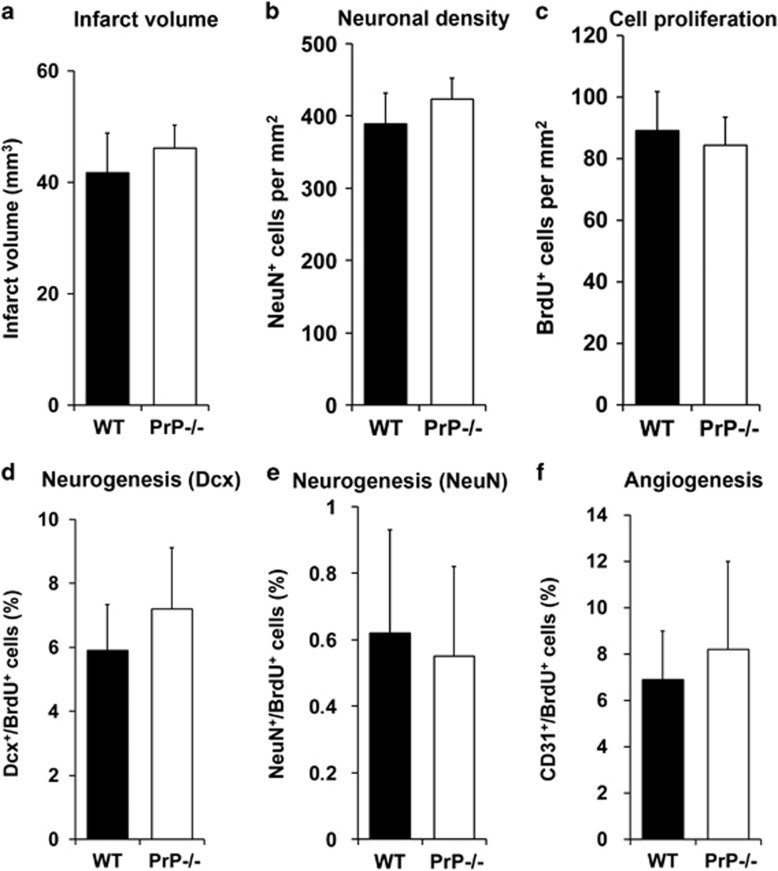
Enhanced post-ischemic neurogenesis and angiogenesis in PrP−/− mice is a consequence of increased infarct size. (**a**) Infarct volume determined by TTC staining, (**b**) neuronal density in the striatum determined by NeuN immunohistochemistry, (**c**) cell proliferation measured by BrdU immunolabeling, (**d**) neurogenesis evaluated by co-labeling of the immature neuronal marker Dcx and BrdU, (**e**) neurogenesis assessed by co-labeling of the mature neuronal marker NeuN and BrdU, and (**f**) angiogenesis examined by co-labeling of the endothelial marker CD31 and BrdU in WT mice exposed to 45 min MCA occlusion and PrP−/− mice exposed to 30 min MCA occlusion followed by 24 h reperfusion (**a**) or 28 days reperfusion (**b**–**f**). Note that in the presence of very similar brain injury (**a** and **b**), post-ischemic cell proliferation (**c**), neurogenesis (**d**–**e**) and angiogenesis (**f**) do not differ between WT and PrP−/− mice

Neurogenesis and angiogenesis are stimulated upon focal cerebral ischemia in a way that closely depends on the severity of brain injury.^5, 25^ To first evaluate whether enhanced neurogenesis and angiogenesis were a consequence of exacerbated brain damage only, we exposed PrP−/− mice to a shorter MCA occlusion of 30 min, whereas WT mice again were submitted to 45 min of MCA occlusion. Analysis of infarct volume at 24 h post-stroke and of neuronal density at 28 day post-stroke revealed a similar extent of brain injury in both groups (Figures 4a and b). Analysis of post-ischemic neurogenesis and angiogenesis on day 28 revealed no significant difference in cell proliferation and cell differentiation (Figures 4c and f). The number of BrdU^+^, Dcx^+^/BrdU^+^, NeuN^+^/BrdU^+^ and CD31^+^/BrdU^+^ cells was very similar in both groups.

The aforementioned observations were further backed up by inducing a 75-min stroke in WT mice, which was associated with a similar (enhanced) extent of brain injury compared with a 45-min stroke in PrP−/− mice. We observed an infarct volume of 62.4±8.1 mm^3^ after 24 h and a neuronal density of 297.1±28.5 NeuN^+^ cells per mm^2^ on day 28 in these WT mice. The latter also developed increased amounts of BrdU^+^ cells (121.7±9.4 per mm^2^) and increased proportions of Dcx^+^/BrdU^+^ (12.4±2.0%) and NeuN^+^/BrdU^+^ (1.7±0.39%) cells, which were on the same order as in PrP−/− mice exposed to a 45-min stroke. These data suggest that the increased neurogenesis and angiogenesis in PrP−/− mice is a consequence of increased brain injury.

### PrP^c^ induces post-ischemic neuroprotection via inhibition of proteasome-induced hypoxia-inducible factor 1*α* degradation

**Figure 5:**
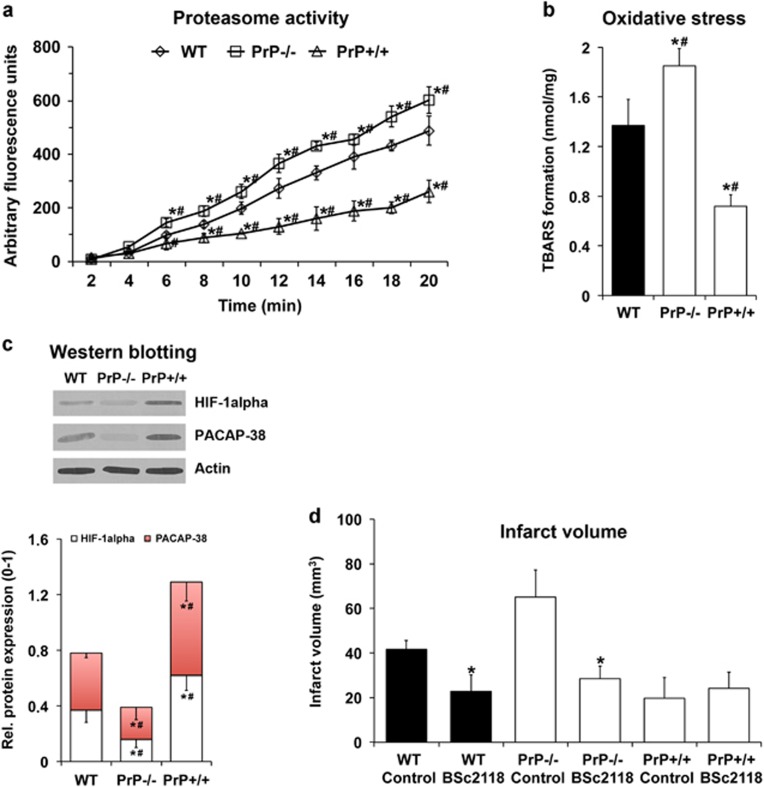
Proteasome-mediated HIF-1*α* stabilization initiates PrP^c^-induced neuroprotection. (**a**) Proteasomal activity analyzed by Suc-LLVY-AMC cleavage, (**b**) oxidative stress determined by thiobarbituric acid reactive substances (TBARS) analysis, (**c**) HIF-1*α* and PACAP-38 abundance evaluated by western blotting and (**d**) infarct volume assessed by TTC staining of WT, PrP−/− and PrP+/+ mice exposed to 45 min MCA occlusion followed by 24 h reperfusion. For studies in (**a**–**c**), tissue samples were obtained from left ischemic hemispheres, and western blots in (**c**) were normalized with optic densities measured in *β*-actin blots (representative western blots are also shown). For studies in (**d**), animals had intrastriatally been pretreated with the solvent DMSO (control) or with the proteasome inhibitor BSc2118 12 h before MCA occlusion. *Significantly different from WT mice (**a**–**c**) or from corresponding control mice (**d**), *P*<0.05. ^#^Significantly different from PrP−/− or PrP+/+ mice, *P*<0.05

We have previously shown that overexpression of PrP^c^ results reduces phosphorylation of ERK-1/2 after focal cerebral ischemia in mice.^23^ ERK-1/2 is activated by the proteasome,^26, 27, 28^ which aggravates post-ischemic brain injury via increased oxidative stress and hypoxia-inducible factor 1*α* (HIF-1*α*) degradation.^29^ To elucidate the role of proteasome activation in PrP^c^-induced neuroprotection, we measured proteasomal activity 24 h after 45 min of MCA occlusion. Proteasome activity was significantly increased in PrP−/− mice compared with WT mice, and significantly reduced in PrP+/+ mice compared with WT and, even more so, PrP−/− mice (Figure 5a). Alterations in proteasome activity were associated with changes in oxidative stress, which was significantly increased in PrP−/− mice compared with WT mice and significantly reduced in PrP+/+ mice compared with WT and PrP−/− mice (Figure 5b).

Considering that HIF-1*α* is degraded by the proteasome and inhibition of proteasomal activity reduces ischemic brain injury,^29^ we next evaluated HIF-1*α* abundance in western blots, which was significantly reduced by PrP−/− and significantly increased by PrP+/+ at 24 h post-stroke (Figure 5c). Further taking into consideration that HIF-1*α* upregulates the pituitary adenylate cyclase-activating peptide-38 (PACAP-38), which activates PACAP type 1 receptor (PAC-1), thereby controlling NPC migration and trafficking,^30, 31, 32^ we furthermore determined PACAP-38 abundance in western blots, which was again significantly reduced by PrP−/− and significantly increased by PrP+/+ (Figure 5c).

To verify whether or not PrP^c^-induced post-ischemic neuroprotection involves modification of proteasomal activity, animals received ipsilateral intrastriatal injection of the neuroprotective proteasome inhibitor BSc2118 at 12 h before stroke.^29^ Application of BSc2118 significantly reduced infarct volume at 24 h post-stroke in WT and PrP−/− mice, with no further benefit in PrP+/+ mice (Figure 5d). Interestingly, in PrP−/− mice receiving BSc2118, infarct volume was reduced to levels similar to WT mice. Likewise, analysis of neuronal density on day 28 revealed that BSc2118-induced neuroprotection in both WT (692.4±37.0 *versus* 412.7±24.5 NeuN^+^ cells per mm^2^ in controls) and PrP−/− (718.1±29.7 *versus* 286.9±15.8 NeuN^+^ cells per mm^2^ in controls) mice was stable during the observation period. The extent of brain injury in PrP+/+ mice, however, was again not affected by BSc2118 at this late time point (751.1±61.2 *versus* 704.0±32.9 NeuN^+^ cells per mm^2^ in controls), as has been observed for the acute time point. Of note, pretreatment with BSc2118 also resulted in increased protein expression of HIF-1*α* and PACAP-38 24 h post-stroke in both WT and PrP−/− mice (data not shown). These data indicate that the proteasome is critically involved in the aggravation of brain injury in PrP−/− mice.

### PrP^c^ facilitates peri-infarct homing of grafted NPCs

**Figure 6:**
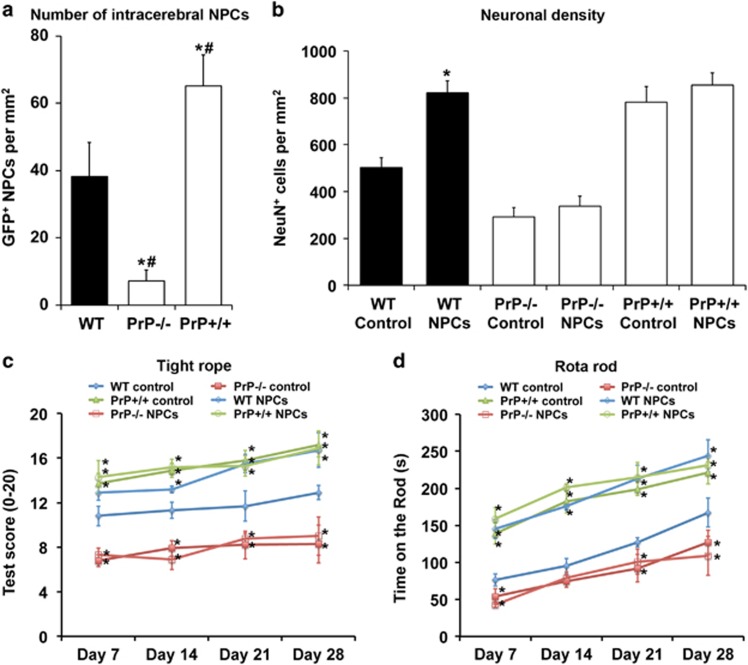
PrP^c^ enables peri-infarct homing of systemically delivered NPCs. (**a**) Density of GFP^+^ NPCs in the ischemic striatum, (**b**) neuronal density in the ischemic striatum revealed by NeuN immunohistochemistry and (**c** and **d**) motor coordination deficits evaluated by tight rope and rota rod tests of WT, PrP−/− and PrP+/+ mice exposed to 45 min MCA occlusion followed by 28 days reperfusion. In (**c** and **d**), mice received intravenous transplantation of normal saline (control) or adult GFP^+^ NPCs (10^6^ cells in 300 *μ*l normal saline) 6 h after reperfusion. *Significantly different from WT mice not receiving NPC treatment, *P*<0.05. ^#^Significantly different from corresponding PrP−/− or PrP+/+ mice, *P*<0.05

Intravenous transplantation of adult NPCs reduces ischemic brain injury and enhances motor coordination recovery.^33, 34^ In light of PrP−/− mice exhibiting reduced PACAP-38 expression, which is critically involved in NPC migration and trafficking, we finally evaluated the effect of PrP^c^ expression on the intracerebral homing of systemically transplanted GFP^+^ NPCs, furthermore examining effects of NPC delivery on ischemic brain injury and neurological recovery. GFP^+^ NPCs were intravenously transplanted in WT, PrP−/− and PrP+/+ mice 6 h after 45 min MCA occlusion. Compared with WT mice, the homing of GFP^+^ NPCs in peri-infarct tissue was significantly reduced in PrP−/− mice and significantly increased in PrP+/+ mice 28 days after stroke (Figure 6a). Pretreatment with BSc2118 significantly increased the number of GFP^+^ NPCs 28 days post-stroke in both WT (58.4±5.7 cells per mm^2^) and PrP−/− (60.2±11.3 cells per mm^2^) mice to levels of PrP+/+ mice (Figure 6a), where the proteasome inhibitor had again no additional effect (data not shown). Notably, the reduced brain accumulation of GFP^+^ NPCs was associated with a lack of neuroprotection (Figure 6b) and functional neurological recovery (Figures 6c and d) induced by NPC transplantation. Likewise, NPC transplantation did not achieve any therapeutic benefit in PrP+/+ mice (Figures 6b and d), which already showed very modest brain injury and motor coordination deficits under control conditions without NPC grafting. These data confirm that PrP^c^ enables NPC migration and pathfinding in the ischemic brain.

## Discussion

Using PrP^c^ wild type (WT), PrP^c^ knockout (PrP−/−) and PrP^c^ overexpressing (PrP+/+) mice exposed to focal cerebral ischemia, we herein show that PrP^c^ induces long-term neuroprotection that persists in the post-acute stroke phase, promoting neurological recovery via mechanisms involving enhanced neurogenesis and angiogenesis. Reduced proteasome activity was noted in the brains of ischemic PrP+/+ mice, which went along with increased HIF-1*α* and PACAP-38 abundance, which are known to control NPC migration and trafficking. On the other hand, proteasome activity was increased in ischemic PrP−/− mice. Pharmacological proteasome inhibition reversed the exacerbation of ischemic brain injury induced by PrP−/−, suggesting that proteasomal deactivation mediates the restorative effects of PrP^c^. PrP^c^ overexpression facilitated the intracerebral homing of systemically administered NPC, which was particularly impeded by PrP−/−.

Previous studies analyzed the effect of PrP^c^ deficiency and overexpression after focal cerebral ischemia, reporting that PrP^c^ deficiency aggravates, whereas PrP^c^ overexpression reduces ischemic brain injury.^20, 21, 22, 23, 24^ Aggravation of ischemic injury by PrP^c^ deficiency was found to involve Akt dephosphorylation, ERK-1/2 phosphorylation and caspase-3 activation.^21, 24^ The precise mechanisms triggering these injurious events were unknown. By demonstrating that PrP^c^ deactivates proteasome activity in ischemic brain tissue and that pharmacological proteasome inhibition reversed the effects of PrP−/− on infarct volume, we now provide such mechanism. ERK-1/2 has previously been found to be activated under conditions of proteasome activation.^26, 27, 28^ Proteasomal activation was found to augment ischemic brain injury via increased oxidative stress and HIF-1*α* degradation.^29^

By comparison of WT, PrP−/− and PrP+/+ mice, we for the first time show that PrP^c^ promotes post-ischemic neurogenesis and angiogenesis. Neurogenesis and angiogenesis are closely linked in the ischemic brain, newly formed vessels providing guidelines for NPCs on their way to the stroke lesion.^6^ Newly formed blood vessels and NPCs mutually stimulate each other via release of trophic factors and guidance molecules.^6, 8, 35^ Neurogenesis and angiogenesis were increased both in ischemic PrP+/+ and in PrP−/− mice. In PrP−/− mice, the increased neurogenesis and angiogenesis were attributed to the exacerbation of brain injury, as revealed in studies in which PrP−/− mice were exposed to a shorter ischemic episode, where cell proliferation and differentiation no more differed between WT and PrP−/− mice. That increased post-stroke neurogenesis in PrP−/− mice does not go along with increased neurological recovery in these animals might appear to be contradictory only at first glance. As a matter of fact, the phenomenon of post-stroke neurogenesis is still under debate as stated afore. Even if new-born cells might positively modulate the post-ischemic milieu via indirect paracrine actions,^33^ this hypothetic beneficial effect might be outweighed by other pro-injurious cascades as, for instance, has been shown in the absence of ephrinB3.^36^ The brain accumulation of systemically delivered NPCs was markedly reduced by PrP−/−, indicating that PrP^c^ deficiency impedes NPC homing and survival.

By providing evidence that HIF-1*α* and its downstream signal PACAP-38 accumulates in ischemic brain tissue under conditions of PrP^c^-induced proteasome deactivation, while HIF-1*α* is degraded as a consequence of PrP^c^ deficiency-induced proteasome overactivation, we identified a possible mechanism for the PrP^c^-induced neurogenesis and angiogenesis. Pharmacological proteasome inhibition has previously been described to enhance post-ischemic neurological recovery, neurogenesis and angiogenesis via mechanisms involving HIF-1*α* accumulation.^29^ HIF-1*α* controls NPC migration and trafficking via PACAP-38,^30, 31, 32^ which is *de novo* expressed in the ischemic brain^37^ and itself promotes post-ischemic neurological recovery and neuronal survival.^38^ These observations are in line with Lee *et al.* who demonstrated increased post-ischemic levels of stress-inducible protein-1 (STI-1), which is a ligand of PrP^c^, via binding of HIF-1*α* to the STI-1 promoter, thus yielding enhanced homing of bone marrow-derived cells and neurological recovery.^39^ However, one has to keep in mind that despite the proteasome (and HIF-1*α*) being critically involved in the present work, the proteasomal activity certainly affects a plethora of cell cascades that are not exclusively analyzed herein.

By demonstrating that PrP^c^ overexpression markedly enhances the intracerebral homing of systemically delivered adult NPCs, we finally confirmed that PrP^c^ promotes NPC migration and trafficking. We have previously used the same protocol for the delivery of adult NPCs, demonstrating that adult NPCs promote neurological recovery after focal cerebral ischemia.^33, 34^ In line with these earlier studies, NPC transplantation reduced neuronal injury and neurological deficits in ischemic WT mice, but failed to show any benefits in PrP−/− or PrP+/+ mice. While the lack of effects in PrP−/− mice may be a consequence of low intracerebral numbers of grafted NPCs, the lack of effects in PrP+/+ mice might rather represent a ceiling effect in animals exhibiting very subtle injury already under control conditions. Based on the presented data here, the PrP^c^–proteasome–HIF-1*α* link might represent a promising target for restorative stroke therapies.

## Materials and Methods

### Experimental design

Experiments following the ARRIVE criteria were conducted in compliance with institutional guidelines and approved by local government authorities. All mice were strictly randomized, experimenters being blinded to experimental conditions. Male WT, PrP−/− and PrP+/+ mice (24–27 g)^23, 24, 40^ were kept under controlled circadian rhythm with free access to food and water. Mice were exposed to transient focal cerebral ischemia followed by 24 h or 28 days reperfusion. Survival rates were 100% for 24 h reperfusion and ranged from 81.3 to 100.0% for 28 days reperfusion (see Supplementary Table S1). For assessment of post-ischemic neurogenesis and angiogenesis on day 28, mice received daily single intraperitoneal (i.p.) injections of bromodeoxyuridine (BrdU; Sigma-Aldrich, Taufkirchen, Germany; 50 mg/kg) on days 8–18 (see Supplementary Figure S1 for details of experimental protocol).

### Induction of transient focal cerebral ischemia

Mice were exposed to transient focal cerebral ischemia using the intraluminal occlusion model as previously described by our group.^29^ Animals were deeply anesthetized with isoflurane (1–1.5%) in 30% O_2_ (remainder N_2_O). After a neck midline incision, the left common carotid artery (CCA) was prepared and a silicon-coated monofilament (Doccol, Sharon, MA, USA) was inserted into the left CCA. The filament was gently moved forward until the offspring of the MCA. Thereafter, the filament was kept *in situ* for 45 min (for 30 min or 75 min in some experiments) under constant laser Doppler flowmetry, using a flexible probe attached to the skull overlying the left MCA territory. After filament removal, cerebral blood flow was monitored for additional 15 min in order to ensure adequate reperfusion. Rectal temperature was kept constantly at 36.5–37.0 °C using a rectal probe attached to a feedback heating system.

### Preparation of NPCs and cell transplantation

Adult SVZ-derived NPCs were generated as previously described.^33^ Preparations were done using male green fluorescence protein (GFP) transgenic mice (C57BL/6-Tg ACTB-enhanced green fluorescence protein, 1Osb/J; JAX Laboratory, Bar Harbor, ME, USA, 6–8 weeks old), which carry the GFP gene under an actin promoter. For all experiments, GFP^+^ NPCs from passages 3 to 8 were used. NPCs (10^6^ cells dissolved in 300 *μ*l normal saline) were grafted 6 h after stroke by intravenous infusion over 10 min. Control animals received normal saline infusions. Animals were killed at 24 h or 28 days post-stroke.

### Stereotactic injection of BSc2118

BSc2118 (30 mg/kg dissolved in 5 *μ*l of 100% dimethyl sulfoxide (DMSO); synthesized and kindly provided by Drs Petra Henklein and Ulrike Kuckelkorn, Institute for Biochemistry, Charité, Berlin, Germany) was injected into the left-sided striatum using a stereotactic frame (injection coordinates 0.4 mm rostral, 3.5 mm ventral and 1.8 mm lateral from bregma) 12 h before stroke in animals anesthetized with ketamine (10 mg/kg) and xylazine (25 mg/kg). Control animals received 100% DMSO injections. Animals were killed at 24 h or 28 days post-stroke.

### Assessment of post-stroke functional neurological recovery

Behavioral tests were performed using animals surviving for 28 days. Animals were trained for the behavioral tests 1–2 days before stroke induction. The tests included the rota rod, tight rope, corner turn and balance beam test as previously described.^41^ In the rota rod test, the time until the animal dropped an accelerating rotating rod was recorded (maximal testing time 300 s), whereas in the tight rope test the animal's ability to cross a tight rope was evaluated using a validated score from 0 (min) to 20 (max). In the corner turn test, the animal was placed in an apparatus consisting of two vertical boards with an angle of 30°. When placed into the corner, a healthy animal randomly leaves the corner to either side, resulting in a laterality index of '0.5'. On the contrary, an ischemic mouse preferentially leaves the corner to the non-impaired body side (i.e. the left side), resulting in a laterality index of up to '1' (indicating most severe neurological impairment). The balance beam test consists of a long beam with constantly reduced width, which was elevated from the ground. Animals had to reach the platform at the end of the beam, and the time until they reached the platform was measured (maximum testing time 60 s). All tests were performed twice on occasion of each time point, and means were calculated for both tests.

### Histological and histochemical analysis

Brain injury at 24 h after stroke was analyzed using 2% 2,3,5-triphenyltetrazolium chloride staining (*n*=6 animals per condition). For this purpose, 2-mm-thick brain slices were obtained, which were evaluated by computer-based infarct volumetry using ImageJ software. Brain injury, cell proliferation, neurogenesis and angiogenesis on day 28 were assessed by immunohistochemistry in 20-*μ*m-thick cryostat sections (*n*=12–15 animals per condition). Three sections per animal were used which were incubated with the following primary antibodies: monoclonal mouse anti-BrdU (1:400; Roche, Basel, Switzerland), monoclonal rat anti-BrdU (1:400; Abcam, Cambridge, UK), polyclonal goat anti-doublecortin (1:50; Santa Cruz Biotechnology, Heidelberg, Germany), monoclonal mouse anti-NeuN (1:1000; Millipore, Darmstadt, Germany), monoclonal rat CD31 (1:200, BD Biosciences, Heidelberg, Germany) and polyclonal rabbit anti-GFP (1:2500; Abcam, UK). Secondary antibodies were as follows: goat anti-mouse Cy-3 (1:400; Dianova, Germany), goat anti-rat Alexa 594 (1:400; Dianova), donkey anti-goat Alexa 488 (1:250; Invitrogen, Carlsbad, CA, USA), goat anti-mouse Alexa 488 (1:100; Jackson ImmunoResearch, Newmarket, UK) and goat anti-rat Alexa 488 (1:250; Invitrogen). Cell densities were evaluated under a fluorescence microscope in a total of four regions of interest in the ischemic striatum as previously described.^41^

### Measurement of thiobarbituric acid reactive substances

During lipid peroxidation thiobarbituric acid reactive substances (TBARS) like malondialdehyde are formed, which react with thiobarbituric acid, giving rise to a chromogenic compound.^33^ Left ischemic hemispheres collected at 24 h post-stroke (*n*=4 animals per condition) were homogenized in cold lysis buffer (50 mmol/l Tris, pH 8.0, 150 mmol/l NaCl, 1% Triton X-100, protease inhibitors). In the homogenates, TBARS formation was photometrically measured at *λ*=532 nm, as described.^33^

### Determination of proteasome activity

The chymotrypsine-like activity of the proteasome was measured in left ischemic hemispheres at 24 h after stroke (*n*=4 animals per condition).^29^ Tissue samples were homogenized in cold lysis buffer containing 100 mM Tris-HCl, 145 mM NaCl, 10 mM ethylenediaminetetraacetic acid and 0.5% Triton X-100 at pH 7.5. Samples were incubated with reaction buffer (50 mM Tris, 20 mM KCl, 2 mM dithiothreitol, 1 mM leupeptin (Sigma-Aldrich) and 1 mM phenylmethylsulfonyl fluoride (Merck, Darmstadt, Germany). As a substrate, Suc-LLVY-AMC (Sigma-Aldrich) was added in a final concentration of 50 *μ*M. Using a fluorescence microtiter plate reader, fluorescence was measured with *λ*_exc._ 355 nm and *λ*_em._ 460 nm. Protease activities were presented as arbitrary units per min per mg of protein. Protein concentration was determined using the Bradford assay.

### Western blots

Tissue samples obtained from left ischemic hemispheres at 24 h after stroke (*n*=4 animals per condition) were homogenized in 50 mmol/l Tris, pH 8.0, 150 mmol/l NaCl, 1% Triton X-100 and protease inhibitors, followed by sodium dodecyl sulfate-polyacrylamide gel electrophoresis using equal amounts of protein. The following primary antibodies were used: polyclonal rabbit anti-HIF-1*α* (Abcam) and polyclonal rabbit PACAP-38 (Calbiochem, Darmstadt, USA). Protein loading was controlled using *β*-actin blots. Membranes were scanned for densitometric analysis of protein abundance.

### Statistics

Data are given as means±standard deviation (S.D.). For comparisons between two groups, Student's *t*-tests were used. For comparisons of multiple groups, one-way analysis of variance followed by the Tukey's *post hoc* tests were performed. *P-*values <0.05 were considered statistically significant.

## Abbreviations

AMC: 7-amino-4-methylcoumarin
ARRIVE: Animal Research: Reporting of *In Vivo* Experiments
Brdu: bromodeoxyuridine
CCA: common carotid artery
Dcx: doublecortin
DMSO: dimethyl sulfoxide
ERK: extracellular signal-regulated kinases
GFP: green fluorescence protein
HIF-1*α*: hypoxia-inducible factor 1*α*
MCA: middle cerebral artery
NPCs: neural progenitor cells
PACAP-38: pituitary adenylate cyclase-activating peptide-38
PrP^c^: cellular prion protein
PrP−/−: PrP^c^ knockout
PrP+/+: PrP^c^ overexpressing
STI-1: stress-inducible protein-1
Suc-LLVY-AMC: SuccSinyl-Leu-Leu-Val-Tyr-AMC
SVZ: subventricular zone
TBARS: thiobarbituric acid reactive substances
TTC: 2,3,5-triphenyltetrazolium chloride
WT: wild type
