# Supplementary material for: Cellular prion protein promotes post-ischemic neuronal survival, angioneurogenesis and enhances neural progenitor cell homing via proteasome inhibition
Source: Cell Death Dis. 2015 Dec 17;6(12):e2024–. doi: 10.1038/cddis.2015.365 (PMC4720898; doi:10.1038/cddis.2015.365)
Supplement: Supplementary Figure 1 [file cddis2015365x1.pdf]

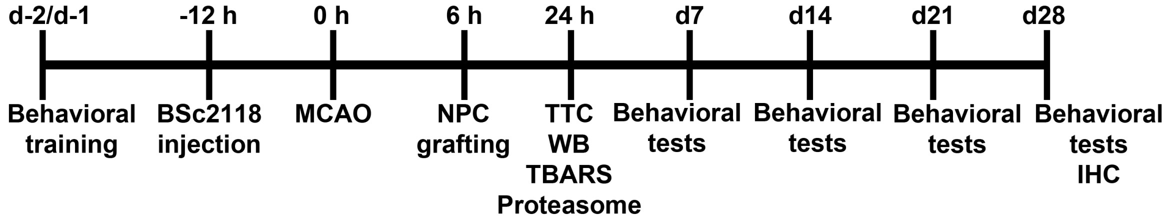

Supplementary Figure S1. Experimental treatment paradigm. Mice were trained 1-2 days before induction of transient focal cerebral ischemia. Some animals received stereotactic intracerebral injection of the proteasome inhibitor BSc2118 (or DMSO as control) 12 h before induction of stroke. At 6 h, some animals received intravenous transplantation of NPCs (or saline as control). Quantitative analyses was performed 24 h and 28 days after stroke induction with additional time points of analysis for assessment of neurological recovery, i.e., days 7, 14 and 21. Abbreviations: IHC, immunohistochemistry; MCAO, middle cerebral artery occlusion; NPC, neural progenitor cell; TBARS, thiobarbituric acid reactive substances; TTC, 2,3,5-triphenyltetrazolium chloride; WB, Western blot
