# Supplementary material for: Cellular prion protein promotes post-ischemic neuronal survival, angioneurogenesis and enhances neural progenitor cell homing via proteasome inhibition
Source: Cell Death Dis. 2015 Dec 17;6(12):e2024–. doi: 10.1038/cddis.2015.365 (PMC4720898; doi:10.1038/cddis.2015.365)
Supplement: Supplementary Table 1 [file cddis2015365x2.pdf]

Supplementary Table S1. Experimental animals used.

	day 1	day 28
**TTC analysis**	n=6 (100%)	n. a.
**Proteasome activity**	n=5 (100%)	n. a.
**TBARS formation**	n=6 (100%)	n. a.
**Western blotting**	n=5-6 (100%)	n. a.
**IHC / Behavioral tests**	n. a.	n=13-16 (81.3-100.0%)

For all experiments, male C57BL6 mice were used that were sacrificed either on day 1 or on day 28. Abbreviations: IHC: immunohistochemistry, TBARS: thiobarbituric acid reactive substances, TTC: 2,3,5-triphenyltetrazolium chloride.
